# Wheat Height Estimation Using LiDAR in Comparison to Ultrasonic Sensor and UAS

**DOI:** 10.3390/s18113731

**Published:** 2018-11-02

**Authors:** Wenan Yuan, Jiating Li, Madhav Bhatta, Yeyin Shi, P. Stephen Baenziger, Yufeng Ge

**Affiliations:** 1 Biological Systems Engineering Department, University of Nebraska–Lincoln, Lincoln, NE 68503, USA; jiatingli@huskers.unl.edu (J.L.); yshi18@unl.edu (Y.S.); yge2@unl.edu (Y.G.); 2 Department of Agronomy and Horticulture, University of Nebraska–Lincoln, Lincoln, NE 68503, USA; madhav.bhatta@huskers.unl.edu (M.B.); pbaenziger1@unl.edu (P.S.B.)

**Keywords:** crop, plant breeding, phenotyping, proximal sensing, remote sensing

## Abstract

As one of the key crop traits, plant height is traditionally evaluated manually, which can be slow, laborious and prone to error. Rapid development of remote and proximal sensing technologies in recent years allows plant height to be estimated in more objective and efficient fashions, while research regarding direct comparisons between different height measurement methods seems to be lagging. In this study, a ground-based multi-sensor phenotyping system equipped with ultrasonic sensors and light detection and ranging (LiDAR) was developed. Canopy heights of 100 wheat plots were estimated five times during a season by the ground phenotyping system and an unmanned aircraft system (UAS), and the results were compared to manual measurements. Overall, LiDAR provided the best results, with a root-mean-square error (RMSE) of 0.05 m and an R^2^ of 0.97. UAS obtained reasonable results with an RMSE of 0.09 m and an R^2^ of 0.91. Ultrasonic sensors did not perform well due to our static measurement style. In conclusion, we suggest LiDAR and UAS are reliable alternative methods for wheat height evaluation.

## 1. Introduction

Plant height is one of the most important parameters for crop selection in a breeding program. For wheat, height is associated with grain yield [1], lodging [2], biomass [3], and resistance to certain disease [4]. Traditionally, plant height is measured manually using a yardstick. This method is labor-intensive and time-consuming when a large number of plants need to be evaluated. In addition, it is prone to error during reading and recording, especially in harsh weather conditions. Alternative but reliable methods for plant height evaluation are needed.

Field phenotyping has been gaining popularity in recent years due to its ability of sensing various crop traits non-destructively in a high-throughput fashion [5,6,7], and sophisticated multi-sensor phenotyping systems such as “Field Scanalyzer” [8], “Ladybird” [9], “Phenomobile” [10] and “Phenomobile Lite” [11] have been reported. As for estimating plant height, several techniques have been adopted in previous research, and the basic principles behind most of the techniques are either time-of-flight (ToF) or triangulation. The ultrasonic sensor, ToF camera [12,13] and most scanning light detection and ranging (LiDAR) techniques are all based on the ToF principle, whereas the structured-light scanner [12], stereo camera or stereo vision [14,15], and structure from motion, which is a technique commonly used in unmanned aircraft system (UAS) imagery, are based on the triangulation principle.

As some of the most common methods for plant height estimation at present, ultrasonic sensor, LiDAR and UAS can be favored over one another because of the unique advantages and disadvantages they possess. The ultrasonic sensor is typically inexpensive and user-friendly, and has a long history of being utilized in plant height measurement [16]. However some of its disadvantages include reduced sensor accuracies when sensors become farther from objects due to the larger field of view (FOV) [17], sensor’s sensitivity to temperature as sound speed changes with temperature [18], and the susceptibility of sound waves to plant leaf size, angle, and surfaces [16]. LiDAR and UAS are relatively new methods for estimating various plant traits such as height, biomass and ground cover [11,19,20,21]. LiDAR is considered a widely-accepted and promising sensor for plant 3D reconstruction because of its high spatial resolution, low beam divergence and versatility regardless of ambient light conditions [9,11,22]. However, LiDAR is also costly, and LiDAR data can be voluminous and challenging to process [23]. UAS has been increasingly used in crop phenotyping over the past decade. A low flight altitude allows images to be captured with relatively high spatial resolution, and it is flexible in terms of temporal resolution and the types of deployed sensors [24,25,26]. However, UAS has limited payload and flight time [10], and the pilot needs to have certain level of proficiency to acquire data with optimal quality.

Ultrasonic sensors, LiDAR and UAS have been exploited for a wide range of crops in the past. However, ultrasonic sensors and UAS were not able to provide consistently accurate height estimations when compared to LiDAR. For example, the ultrasonic sensor has been used to estimate the height of cotton [27,28], alfalfa [29], wild blueberry [30,31], legume-grass [16,32], Bermuda grass [29], barley [33] and wheat [29,34,35], with root-mean-square error (RMSE) from 0.022 to 0.072 and R^2^ from 0.44 to 0.90 reported. Similarly, UAS has been applied to various crops including corn [36,37,38], sorghum [37,39,40] and wheat [20,41,42,43,44,45], and the results from different studies varied greatly, with R^2^ ranging from 0.27 to 0.99. On the other hand, LiDAR has been employed for crops such as cotton [17], blueberry [46] and wheat [8,9,10,11,45,47], and RMSE from 0.017 m to 0.089 m and R^2^ from 0.86 to 0.99 were obtained.

In existing studies of utilizing terrestrial LiDAR, an experimental field is usually scanned by a LiDAR that moves continuously with a constant speed. For a manned multi-sensor system, this might be problematic since sensors such as cameras often require to be stationary to record high quality data, which can cause difficulties for software programming to harness multiple sensor data flows simultaneously, as well as in maintaining the uniform speed during operation. Moreover, despite all the successes and failures of applying ultrasonic sensors, LiDAR and UAS in plant height estimation, a direct comparison between the three methods was missing in previous research. In this study, we aimed to explore a new methodology of processing LiDAR data in the context of a static measurement style, and our ultimate objective was to compare the ultrasonic sensor, LiDAR and UAS in terms of their plant height estimation performance.

## 2. Materials and Methods

### 2.1. Experiment Arrangement

The experiment was conducted during the 2018 growing season at Agronomy Research Farm in Lincoln, NE, USA (40.86027° N, 96.61502° W). The experimental field contained 100 wheat plots where an augmented design with 10 checks replicated twice was used. The wheat lines consisted of 80 wheat genotypes produced at University of Nebraska–Lincoln, NE, USA. The planting was done on 20 October 2017, and the plots were harvested on 29 June 2018.

Five data collection campaigns were conducted during the season. On each occasion, the 100 plots were scanned by a ground phenotyping system and a UAS. The plots were also measured by a yardstick using two methods depending on the growth stage (Table 1). At vegetative stages plant height was measured from soil surface to the top of stem, or apical bud (method A). At reproductive stages plant height was measured from soil surface to the top of spike excluding awns (method B) [1]. For each plot three measurements were taken and averaged as the reference height of the plot. 

### 2.2. Ground Phenotyping System

#### 2.2.1. Hardware

**Figure 1:**
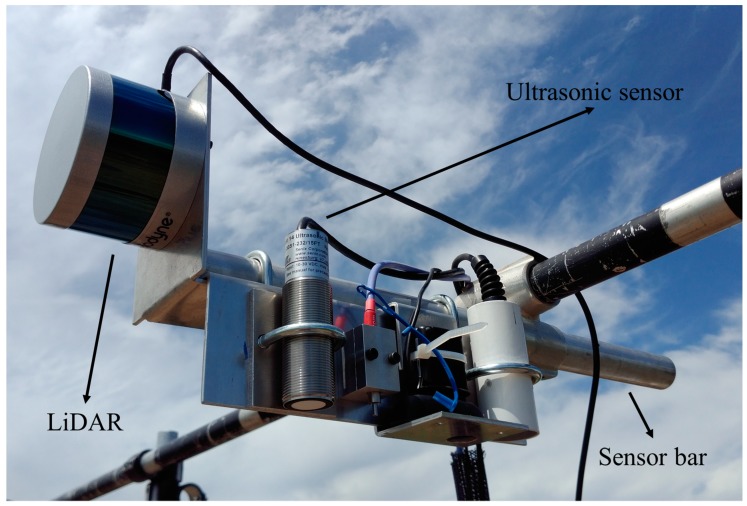
Light detection and ranging (LiDAR) and ultrasonic sensor of the ground phenotyping system.

The ground phenotyping system was built based on the concept of another system developed by Bai et al. [48]. In addition to three ultrasonic sensors (ToughSonic 14, Senix Corporation, Hinesburg, VT, USA) mounted on three sensor bars, a LiDAR (VLP-16 Puck, Velodyne LiDAR Inc., San Jose, CA, USA) was also incorporated on the middle sensor bar (Figure 1).

The ultrasonic sensors have a FOV of 14° and a maximum measurement distance of 4.27 m. The measurement rate was set at 20 Hz. The sensors produce 0 to 10 volts direct current (VDC) signals, which are proportional to the distance between sensors and objects. Voltage signals were measured using a LabJack U6 data acquisition (DAQ) board (LabJack Corporation, Lakewood, CO, USA).

**Figure 2:**
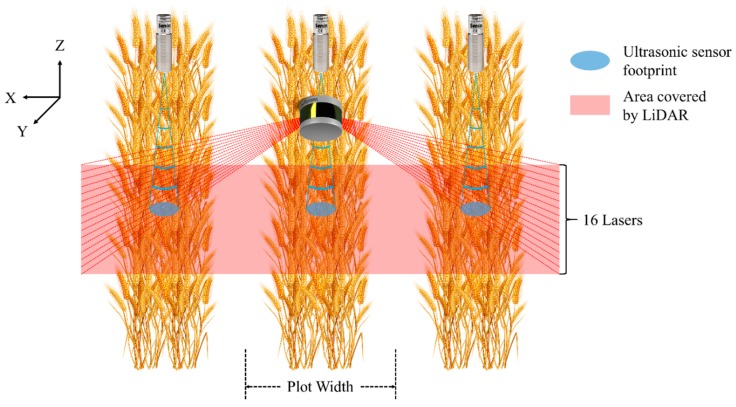
Schematic diagram showing the scanning areas of LiDAR and ultrasonic sensors at each measurement.

The LiDAR transfers data via Ethernet. It has 16 near-infrared lasers with a 903 nm wavelength, and it detects distance up to 100 m. The sensor has a vertical FOV of 30° with a resolution of 2°, and a horizontal FOV of 360° with an adjustable resolution between 0.1° and 0.4°. Since only half of the full azimuth range could be possibly useful for our application of scanning crop canopies (Figure 2), the LiDAR’s horizontal FOV range was configured as 180°, and a 0.1° horizontal resolution was adopted for higher precision. The sensor was also configured to report the strongest return for each laser firing.

#### 2.2.2. Software

**Figure 3:**
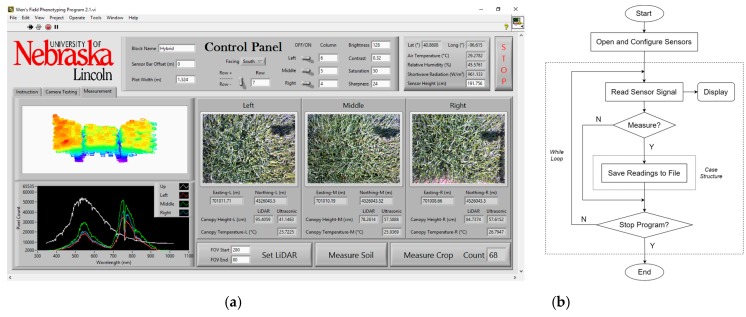
Customized LabVIEW program: (**a**) front panel; (**b**) flowchart of block diagram.

A customized program was developed for sensor controlling and data acquisition using LabVIEW 2016 (National Instruments, Austin, TX, USA) (Figure 3) based on the original program from Bai et al. [48]. The ground phenotyping system adopted a static measurement style [48]. Instead of collecting data continuously, sensor outputs were saved only when designated buttons were triggered.

Voltage signals from ultrasonic sensors were converted to distances in the program through an equation calibrated in lab:

D = 29.116V + 11.641(1)

where D is distance in meters and V is sensor signal in volts. Ultrasonic canopy heights were then calculated as:

H_c_ = H_s_ − D, (2)

where H_c_ is ultrasonic canopy height and H_s_ is ultrasonic sensor height. H_s_ was determined by measuring the distance between the sensors and soil surface before data collection, and LiDAR height was determined in the same way.

**Figure 4:**
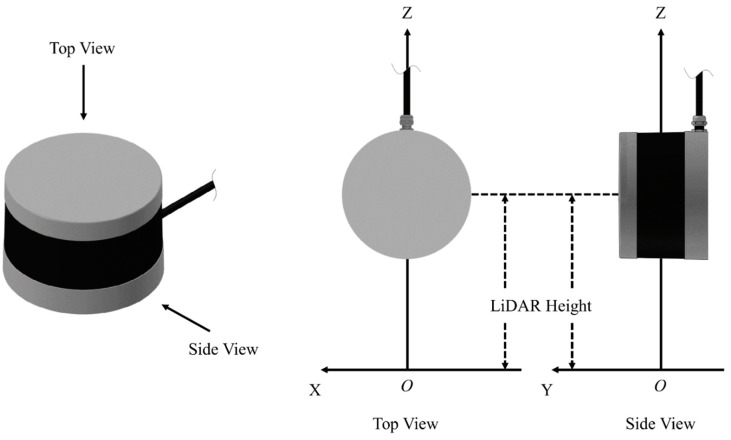
The Cartesian coordinate system for LiDAR point cloud at each measurement.

**Figure 5:**
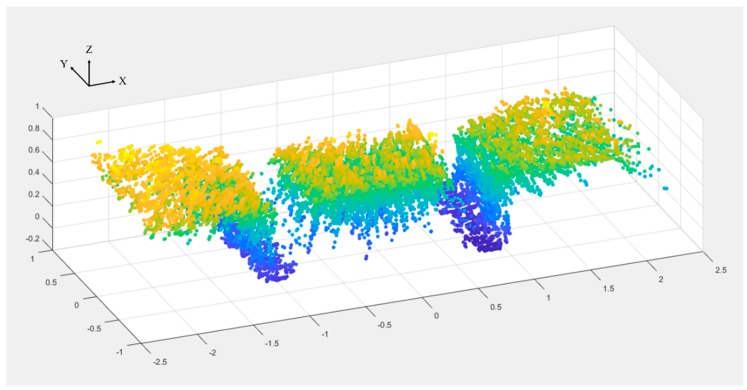
An example of raw LiDAR point cloud at each measurement.

A subprogram was developed for LiDAR and incorporated in the main program. The subprogram receives data packets from LiDAR through the user datagram protocol (UDP). Each data packet contains azimuth and distance information of all 16 lasers, and the subprogram extracts and converts the information into a 3D Cartesian coordinate system. The origin of the coordinate system was defined as shown in Figure 4. After acquiring the XYZ coordinates of the points, the subprogram trims the point cloud in the X-dimension using a threshold of ±1.5 × “plot width” (Figure 2) to delete points outside the desired range. “Plot width” is defined as the distance between the centers of two adjacent alleyways, and was 1.524 m in this study. The point cloud is finally split by two borders of ±0.5 × “plot width” into three parts. Figure 5 is an example of a raw point cloud captured by LiDAR.

#### 2.2.3. Height Extraction from LiDAR Point Clouds

**Figure 6:**
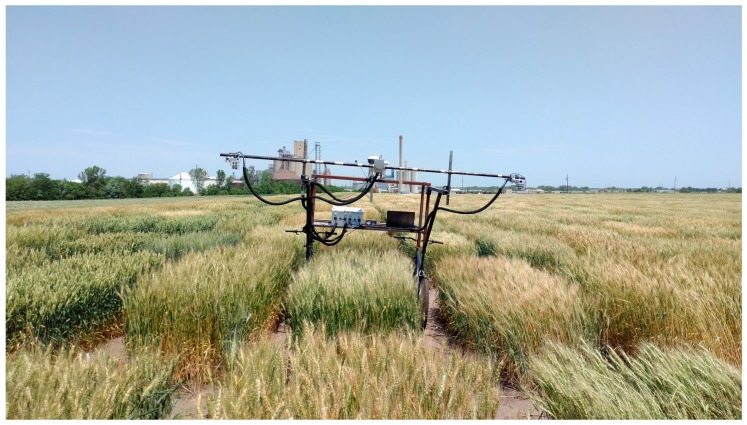
The slanting issue of the phenocart.

One issue that we encountered often in the field was the slant of the phenocart and the sensor bars due to the unevenness and slope of the ground (Figure 6). Corresponding LiDAR point clouds thus would show the tilted angle in the Cartesian coordinate system.

In order to obtain accurate canopy height estimations from LiDAR, pre-processing is necessary for all raw point clouds to correct for this slanting issue before extracting height information. One assumption for pre-processing is that the ground slope variation between the three plots within LiDAR’s horizontal FOV can be ignored. LiDAR point clouds were processed using MATLAB R2017a (The MathWorks, Inc., Natick, MA, USA).

The basic principle of the point cloud pre-processing is that by fitting a linear least-squares curve to the Y-Z plane, the X-Y plane and the X-Z plane of a point cloud, respectively, and converting the slopes of the fitted curves to angles, the tilt of point clouds can be cancelled through rotating point clouds by the magnitude of the angles in reversed direction. For details see Appendix A. After pre-processing was performed, cumulative Z value percentiles of a point cloud with 0.5 percentage intervals from 0 to 100 percent were extracted. In total there were 200 height values extracted and investigated for each plot.

### 2.3. UAS

#### 2.3.1. Hardware

A Zenmuse X5R RGB camera (DJI, Shenzhen, China) was mounted on a rotary-wing unmanned aerial vehicle (UAV), Matrice 600 Pro (M600) (DJI, Shenzhen, China). The RGB camera has an effective pixel resolution of 4608 × 3456. M600 was not available at the 2nd data collection campaign, and was replaced by another rotary-wing UAV, Phantom 3 Pro (P3P) (DJI, Shenzhen, China), with an RGB camera of 4000 × 3000 effective pixel resolution. For both cameras, the capture modes were set as auto, and the white balance was set to Sunny or Cloudy mode based on the specific weather conditions at the data collection campaigns.

#### 2.3.2. Flight Missions

The flight altitude was set to 20 m and 15 m above ground level for M600 and P3P, respectively, to achieve comparable ground sampling distance (GSD). The resulting GSD for M600-derived RGB mosaic was 0.47–0.48 cm/pixel, and was 0.67 cm/pixel for P3P-derived mosaic. The forward overlap and side overlap were both set as 88 percent. 

Twenty-one black and white cross-centered wooden boards, used as ground control points (GCPs), were evenly distributed over the 1.15-hectare field. Their GPS locations were measured by a GNSS RTK-GPS receiver (Topcon Positioning Systems, Inc., Tokyo, Japan), with sub-centimeter accuracy (less than 1 cm) in the X and Y directions, and centimeter accuracy (less than 2 cm) in the Z direction.

#### 2.3.3. Image Processing

RGB images were processed using Pix4Dmapper (Pix4D, Lausanne, Switzerland) to generate a digital surface model (DSM) in three steps: initial processing (step 1), point cloud and mesh (step 2), and DSM, orthomosaic and index (step 3). In step 1, 2D key-points—points with common features among several images—were matched, and 3D automatic tie points were derived. To further geo-calibrate the images, the geo-coordinates of GCPs’ centers were imported and marked out in associated images. In step 2, additional tie points were added to generate a densified point cloud based on the automatic tie points. In step 3, Delaunay triangulation was used to interpolate between tie points to generate the DSM, and the output was saved as a GeoTIFF file.

**Figure 7:**
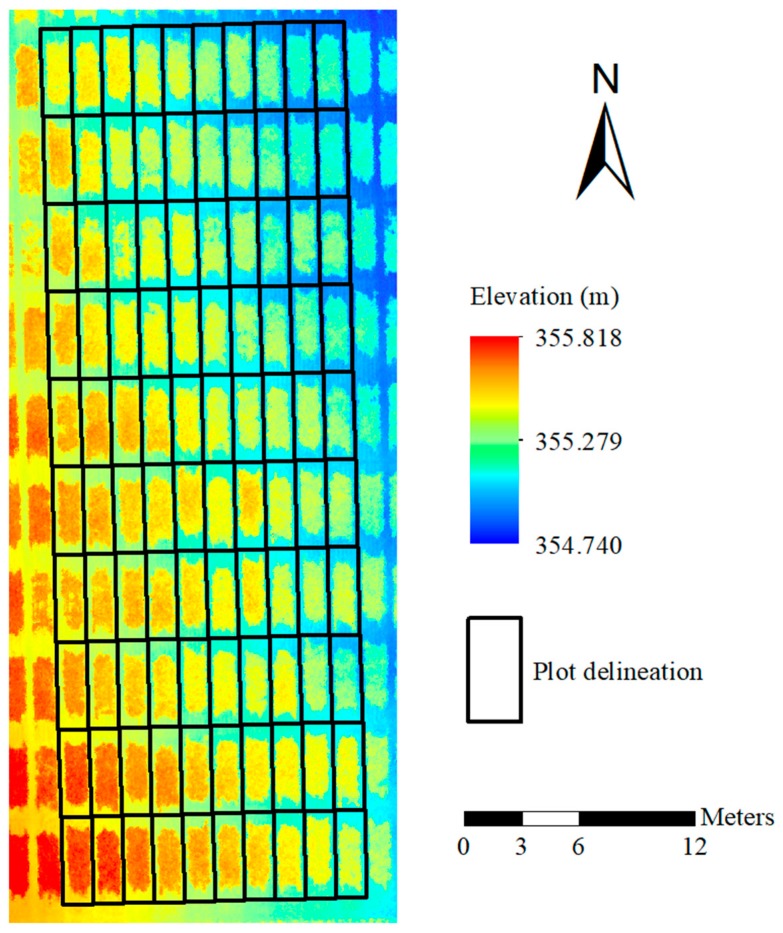
Digital surface model (DSM) map of the investigated 100 plots with plot delineation.

Since manual measurements represented the average heights of plots, UAS-derived plant heights were calculated on a plot level. The 100 plots were equally delineated in a shapefile in ArcMap (ArcGIS v10.5.1, Environmental System Research Institute Inc., Redlands, CA, USA) as shown in Figure 7. Each black rectangle was matched with the actual wheat plot by a designated ID number.

#### 2.3.4. Plant Height Extraction

A plant height map was created by subtracting a digital terrain model (DTM) from the DSM. DTM represents the elevation of bare soil, and it was generated by an interpolation tool, Kriging, in ArcGIS. Roughly 40% of all soil pixels were randomly selected from the DSM map for the interpolation. In order to explore the most representative plant height for each plot, pixel value percentiles within each plot delineation with 1 percentage intervals from 0 to 100 percent were calculated.

## 3. Results

### 3.1. Raw Point Clouds versus Processed Point Clouds

To evaluate the effectiveness of LiDAR point cloud pre-processing, plant heights were also extracted from all raw point clouds. With manual measurements being the standard, the minimum RMSE and the corresponding percentile of raw point clouds and processed point clouds at each data collection campaign were compared (Table 2).

The point cloud pre-processing consistently improved the precision of LiDAR’s plant height estimation by lowering the minimum RMSE at different data collection campaigns by between 12.85% and 44.95%, which confirmed its effectiveness for reducing the influence of the uneven ground surface on point clouds.

### 3.2. LiDAR Height Estimation Performace by Date, Manual Method and Plot Position

**Figure 8:**
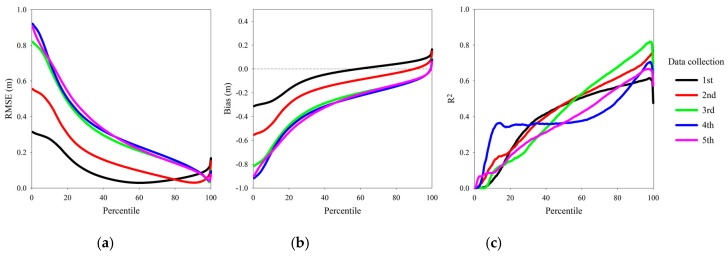
Statistical results of heights extracted at different percentiles from processed LiDAR point clouds over five data collection campaigns: (**a**) RMSE; (**b**) bias; (**c**) R^2^.

By comparing to manual measurements, RMSE, bias and R^2^ of the heights extracted at each of the 200 percentiles of the processed point clouds across five data collection campaigns were investigated (Figure 8).

For a point cloud, low percentiles of the Z value represent the height of ground, and high percentiles represent the height of vegetation above ground. Since the height of a wheat plot was never measured as the height of the tallest plant, it can be seen why RMSE dropped as percentile increased and rose again when percentile approached 100 percent. At the percentiles of the minimum RMSE, the average bias over five data collections was −0.0011 m, which demonstrated LiDAR’s accuracy. The percentiles for maximum R^2^ fluctuated between 98 and 99 percent, which did not appear to agree with the percentiles of minimum RMSE for the first two data collection campaigns (Table 2).

Considering that the percentile of minimum RMSE could always vary if data were collected at different dates, identifying the optimal percentile for each individual data collection campaign was impractical. Instead of treating all data collection campaigns equally and choosing one universal percentile, we classified the 1st and 2nd data collection campaigns as the method A category, and the 3rd, 4th and 5th data collection campaigns as the method B category (Table 1) for more precise height estimations. The RMSE of method A, method B and the all category (meaning all five data collection campaigns were treated as a whole) were compared (Table 3).

The effect of plot position on RMSE was also investigated (Table 3). LiDAR had a fixed horizontal resolution, so the closer an object was to LiDAR, the denser the acquired point cloud of that object would be. In our case, the point cloud generated at each measurement included two side plots and one middle plot, with LiDAR positioned above the middle plot; thus, middle plots had denser point clouds than side plots. On average the point clouds of side plots had about 6000 points while those of middle plots had about 8000 points.

Based on Table 3, the manual method affected RMSE substantially as the minimum RMSE of the all category was 37.45% and 65.08% higher than the minimum RMSE of method A and B categories, respectively. Thus, it makes sense to use different optimal percentiles for the two method categories for future work. However, plot position did not seem to affect RMSE in a significant way, with an average RMSE increase of 0.0026 m when plot positions were not differentiated in the two method categories. Hence, the effect of plot position can be ignored in the future as the additional RMSE impact should be minor.

### 3.3. Optimal Pixel Value Percentiles of Plant Height Map from UAS

Using manual measurements as the reference, RMSE, bias and R^2^ of plant heights extracted at each of the 100 pixel value percentiles of the plant height map were investigated. With the same reasoning as mentioned in Section 3.2, method categories were also applied here. For the method A category, the 89th percentile provided the smallest RMSE, of 0.0439 m, with a bias of −0.0035 m and an R^2^ of 0.897. For the method B category, the 100th percentile achieved the lowest RMSE of 0.1086 m, and the bias and R^2^ were −0.0702 m and 0.436, respectively.

### 3.4. Height Estimation Comparison between LiDAR, Ultrasonic Sensor and UAS

**Figure 9:**
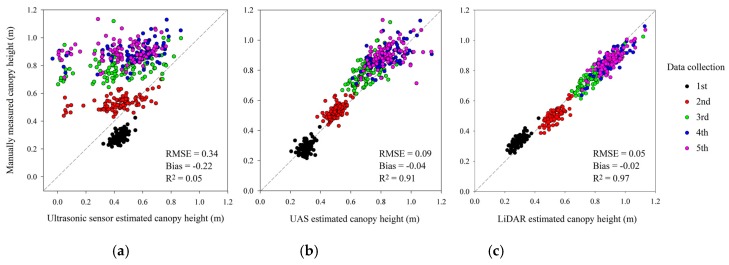
Manually measured canopy heights versus instrument estimated canopy heights: (**a**) ultrasonic sensors; (**b**) UAS; (**c**) LiDAR.

Over five data collection campaigns, ultrasonic sensor estimated canopy heights, UAS estimated canopy heights where 89th and 100th pixel value percentiles were chosen for method A and B categories, and LiDAR estimated canopy heights where 82nd and 99th Z value percentiles of processed point clouds were chosen for method A and B categories were plotted against manual measurements (Figure 9).

Among the three methods, LiDAR performed the best, UAS provided reasonable results, and ultrasonic sensors did not achieve suitable height estimates. With a large RMSE of 0.34 m and a low R^2^ of 0.05, ultrasonic sensors tended to overestimate wheat canopy heights during the 1st data collection campaign and underestimate heights in the remaining data collection campaigns. As discussed in Section 4.1, ultrasonic sensors also provided some negative readings. Overall, UAS provided good wheat heights estimates, with an RMSE of 0.09 m and an R^2^ of 0.91. However, this method tended to underestimate heights and its estimations tended to scatter more as wheat plants grew taller. LiDAR provided the most precise and accurate height estimations throughout the season, with a low RMSE of 0.05 m, a low bias of −0.02 m and a high R^2^ of 0.97. In terms of the results, LiDAR and UAS can be considered as alternative plant height evaluation methods.

## 4. Discussion

### 4.1. Ultrasonic Sensor

The poor performance of ultrasonic sensors in this study can be explained by sensor limitations, wheat morphology and our measurement style. An ultrasonic sensor generates sound waves to detect distance. When the sound waves are not reflected straight back to the sensor, due to either sensor orientation or object surface orientation, the ultrasonic sensor may not capture the reflected sound waves. In this study, the slanting issue of the phenocart could be a source of such a problem. Further, when the surface of an object is not large enough to create strong echoes, an ultrasonic sensor may not treat the weak echoes as valid signals. A typical wheat plant has narrow leaves and thin spikes, thus making it hard for ultrasonic sensors to detect valid signals reflected from wheat. Moreover, because of our static measurement style, for each plot the ultrasonic sensor was only able to sample a small area (about 0.05 m^2^ assuming 1 m distance between sensor and canopy) to represent the whole plot. Due to within-plot variation, the random error from sampling could not be assessed or corrected, which led to the low performance of the ultrasonic sensors. Andújar et al. [35] also used ultrasonic sensors in a static measurement style to detect weeds among wheat plants, and a low Pearson’s correlation of 0.32 between ultrasonic sensor readings and manually measured wheat heights was observed.

**Figure 10:**
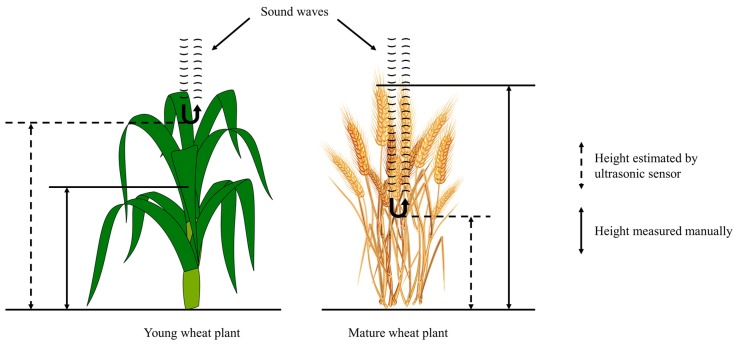
Two scenarios where ultrasonic sensor estimations disagree with manual measurements.

The overestimation and underestimation of wheat height by ultrasonic sensors is illustrated in Figure 10. For a young wheat plant, clustered leaves with natural curvature appeared to reflect sound waves effectively, but the reference height was measured as the height of the stem top instead of the leaf top (method A). As wheat plants grew taller and spikes started to emerge, only the vegetation at the bottom of the plant seemed to have sufficient density to reflect strong echoes, hence resulting in a spike tip height that was lower than that found by manual measurement (method B).

Near-zero canopy heights can appear when ultrasonic sensors cannot detect any significant echoes except for those reflected from ground. Moreover, if the phenocart is slanted so that the distance between ultrasonic sensors and ground at a given moment is larger than H_s_ in Equation (2), negative canopy heights will be recorded.

To improve plant height estimation of ultrasonic sensors, a continuous measurement style—i.e., multiple measurements per plot—is preferred. In a previous study by Scotford and Miller [34], approximately 180 wheat height measurements from ultrasonic sensor were recorded for each plot, and it was found that the 90% percentile of each data set provided the best wheat height estimation, with the lowest RMSE for a wheat variety of 0.046 m. Pittman et al. [29] extracted 25–30 ultrasonic sensor readings per wheat plot, and found a Pearson’s R of 0.85 compared to manual measurements. 

The continuous measurement style is superior to static measurement in terms of obtaining better ultrasonic height estimations. In the context of our manned multi-sensor system, however, the phenocart was often required to stop to capture images. Two issues could occur if a continuous measurement style were adopted for the system: first, due to the highly variable phenocart speed on a field with a rough surface, inconsistent numbers of height measurements could be recorded for different plots; second, a large number of repeated measurements will be taken from the same sampling area when the phenocart is stationary. Both issues can bias the data and make them troublesome to process. The static measurement style may, therefore, still be preferable for our system, in which case the ultrasonic sensor is not the best method for wheat height estimation.

### 4.2. UAS

In this study, UAS tended to underestimate wheat canopy heights, and the underestimation became more significant after the 2nd data collection campaign. Other related studies also found similar issues [41,43]. One possible explanation is that, due to the resolution limitation of cameras, wheat spikes could not be effectively detected in the images. The plant height map could only represent the heights of wheat leaf tops that were clearly identifiable in the images instead of the heights of wheat spike tips.

Scattered UAS plant height estimations near the end of the season might be explained by wind during data collection. Wind would not affect manual measurements as plants were held by hand while measuring; however wind could cause large movement of plants during UAS data collection, which became worse when plants were taller. Inconsistent plant positions among images could reduce mosaic quality, thus leading to higher error when generating DSM.

Generally, UAS-derived plant heights are obtained from the difference between DSM and DTM, and methods of deriving DTM vary among studies. Interpolating soil points segmented from DSM [43,45] and scanning bare soil before seedling emergence [43,44] have been indicated to provide similar plant height estimations [43]. Typically, a specific pixel value within each plot delineation on a plant height map is selected to represent plant height of each plot, such as the average [41,43,44], the 99.5th percentile [45] or the 100th percentile [49]. However, these values might not necessarily be the most representative for plant height depending on the growth stage, thus 100 different pixel value percentiles from the plant height map were investigated in this study.

The 0.91 R^2^ achieved in this study was not better than those of other relevant studies on wheat, such as an R^2^ of 0.92 from Bendig et al. [41], R of 0.88 to 0.98 from Schirrmann et al. [44], and an R^2^ of 0.99 and an RMSE less than 0.03 m at a single data collection campaign by Holman et al. [43]. Considering UAS plant height estimations are also affected by factors including image resolution, sample size and plant growth stage, the pixel value percentile selection methodology used in this study can, nonetheless, serve as a reference for future research.

To improve UAS plant height estimation in future studies, higher spatial resolution of images can be achieved by decreasing flight altitude, which will, however, increase fight time. Also, point clouds can be directly used instead of by extracting plant heights from a rasterized 2D plant height map, thus reserving the greatest quantity of plant 3D information.

### 4.3. LiDAR

The LiDAR point cloud pre-processing proposed in this study effectively reduced the influence from the slanting issue of the phenocart on the field. However, when ground is fully covered by vegetation, LiDAR with strongest return mode might not capture enough ground points, and pre-processing of the point cloud could not be undertaken. Due to the beam divergence of the lasers, a single firing of a laser can hit multiple objects resulting in multiple returns, and, typically, LiDAR can be configured to report multiple returns. A suggested solution is to configure LiDAR in multiple return mode since the last return signal has a higher chance of being reflected by soil, so a sufficient amount of ground points might be collected.

For processed point clouds, the minimum RMSE and the corresponding percentile increased as wheat grew taller (Table 2). As method B was measuring the tip of wheat spikes while method A was measuring the top of wheat stems, it was expected that the optimal percentiles increased with data collection campaigns. Wind was suspected to be the reason for the increasing RMSE. As wheat plants get taller, wind can cause a larger degree of bending in plants, and LiDAR can capture deformed point clouds due to the wind. At the 5th data collection campaign, when the minimum RMSE was the largest, the wind speed on the field was maintained at 8.0 to 8.9 m/s, with gust speeds up to 14.8 m/s.

Generally, extracting plant heights from point clouds can include the following steps: soil level estimation, noisy point removal, rasterization of the point cloud, and percentile selection. Similar to the purpose of our ground baseline correction (Appendix A.4), most studies removed the effect of uneven soil levels by subtracting the corresponding soil height from vegetation points. The peak of the point cloud’s Z value histogram [11,45], mean height of non-vegetation points [10], vehicle wheel contact points [9] and direct soil measurement at the beginning of the season [47] have all been used to estimate soil level. Some studies have also assumed constant distance between sensor and ground [17]. LiDAR can detect spurious points in very bright light conditions [11], and some studies [11,45] removed outlier points by the method proposed by Rusu et al. [50]. We did not perform any noise removal technique, since even if a small number of erroneous points existed, they would not affect our optimal percentile significantly. Point clouds are sometimes rasterized for easier future data analysis, and statistics such as maximum, mean and certain percentiles are calculated for each grid or pixel. We preferred point clouds over 2D height maps because rasterization can cause loss of information. “Percentiles” of point clouds are essentially plant heights, and 95th [10], 95.5th [11], 99.5th [45] and 100th percentiles [17,46] have all been adopted in different studies.

Compared to the results of other relevant studies on wheat height estimation using LiDAR, such as an R^2^ of 0.90 and an RMSE of 3.47 cm from Madec et al. [45], R^2^ of 0.88 and 0.95 at two different months from Underwood et al. [9], an R^2^ of 0.993 and an RMSE of 0.017 m from Jimenez-Berni et al. [11], and an R^2^ of 0.86 and an RMSE of 78.93 mm from Deery et al. [10], this study demonstrated the practicality of obtaining adequate wheat canopy height estimations using LiDAR based only on a section of a plot instead of the whole plot. The advantage here was higher system throughput and easier data processing, but the downside might be lower precision for plant height estimation. In this study, the advantage of 3D LiDAR technology allowed us to adopt a static measurement style, whereas for a 2D LiDAR, the continuous motion of the sensor is a necessity for generating 3D point clouds.

Compared to an ultrasonic sensor, LiDAR had a much higher spatial resolution, and the laser beams were thin and diverged much less than sound waves. Compared to UAS, LiDAR point clouds were direct measurements, while the plant height map derived from UAS images was an indirect measurement. Thus, LiDAR’s overall superior results were expected. The better performance of LiDAR compared to UAS also showed the advantage of proximal sensing over remote sensing; however, in terms of system throughput, it was difficult for a ground system to match with UAS: our ground phenotyping system normally took less than 15 min to scan 100 plots, whereas UAS could cover the same area within 6 min.

To improve LiDAR’s plant height estimation performance, in the context of our static measurement style, denser point clouds—i.e., collecting more data packets—might provide more consistent results. In this study, due to the insufficient number of data collection campaigns, our data did not cover all the important growth stages, so we were, thus, unable to categorize data collection campaigns by growth stage. For future work optimal percentiles at each growth stage of wheat can be further investigated and established, which should provide more precise and accurate plant height estimations.

## 5. Conclusions

In this study, our proposed LiDAR point cloud pre-processing was demonstrated to be effective at reducing the influence of an uneven ground surface, and a LiDAR point cloud generated from a section of a plot was proven to be sufficient for providing precise and accurate plant height estimates. This methodology can be a reference for future studies that wish to adopt a static measurement style. With the reasonable results from UAS obtained in this study, considering the high-throughput for data collection, UAS can be a promising height estimation tool for a wide range of plants. The ultrasonic sensor, when used for plant height estimation in a static measurement style, is not suggested for plants with tall sward structures, such as mature wheat plants. In conclusion, LiDAR and UAS are both recommended as reliable alternative methods for wheat height evaluation.

## Figures and Tables

**Table 1:** Data collection campaign dates of manual measurement, the ground system and the unmanned aircraft system (UAS) for wheat height evaluation.

Data Collection Campaign	Growth Stage	Manual		Ground System	UAS
		Date	Method	Date	Date
1st	Jointing stage: Feekes 6	7 May	A	7 May	7 May
2nd	Flag leaf stage: Feekes 8	15 May	A	15 May	15 May
3rd	Boot stage: Feekes 9	23 May	B	23 May	21 May
4th	Grain filling period: Feekes 10.5.3	31 May	B	31 May	1 June
5th	Physiological maturity: Feekes 11	16 June	B	15 June	18 June

**Table 2:** Optimal root-mean-square error (RMSE) and percentile of raw and processed point clouds at each data collection campaign.

Data Collection Campaign		1st	2nd	3rd	4th	5th
Raw Point Clouds	Minimum RMSE (m)	0.0462	0.0389	0.0643	0.0467	0.0521
	Optimal Percentile	67.5th	85th	99.5th	99th	99.5th
Processed Point Clouds	Minimum RMSE (m)	0.0290	0.0300	0.0354	0.0407	0.0420
	Optimal Percentile	60th	91st	99th	99th	99.5th

**Table 3:** Effects of manual method and plot position on minimum RMSE of processed LiDAR point clouds.

Category	Method A		Method B		All	
Number of Plots	200		300		500	
Minimum RMSE (m)	0.0478		0.0398		0.0657	
Optimal Percentile	82nd		99th		98th	
**Sub-Category**	**Side**	**Middle**	**Side**	**Middle**	**Side**	**Middle**
Number of Plots	140	60	200	100	340	160
Minimum RMSE (m)	0.0436	0.0491	0.0395	0.0327	0.0649	0.0624
Optimal Percentile	77th	89th	99th	99.5th	97th	99th

**Figure A1:**
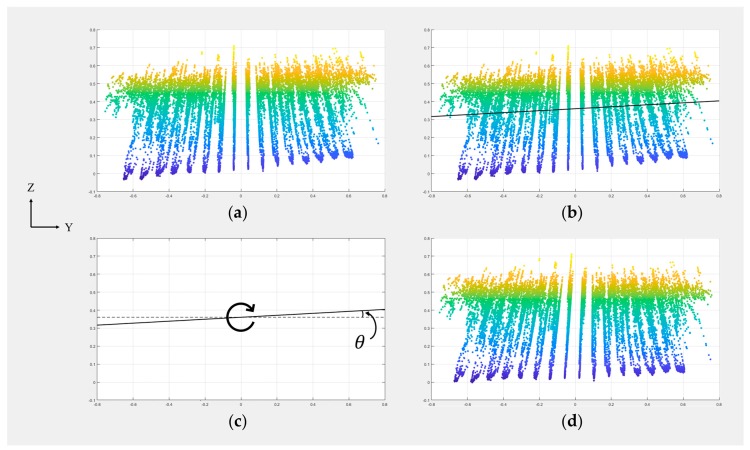
An example of Y-Z plane rotation correction: (**a**) Point cloud before rotation; (**b**) Fit a linear curve to points on Y-Z plane; (**c**) Rotate points on Y-Z plane by the angle θ; (**d**) Point cloud after rotation.

**Figure A2:**
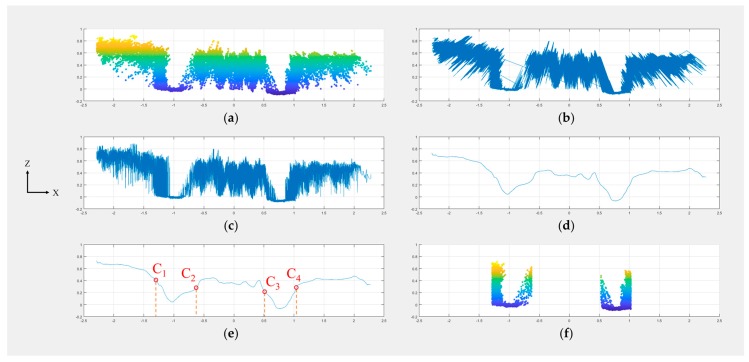
An example of extracting coarse alleyway point clouds: (**a**) point cloud before rotation; (**b**) line graph before sorting; (**c**) line graph after sorting; (**d**) smoothed line; (**e**) positions of the four most significant changes; (**f**) deletion of points beyond the desired range.

**Figure A3:**
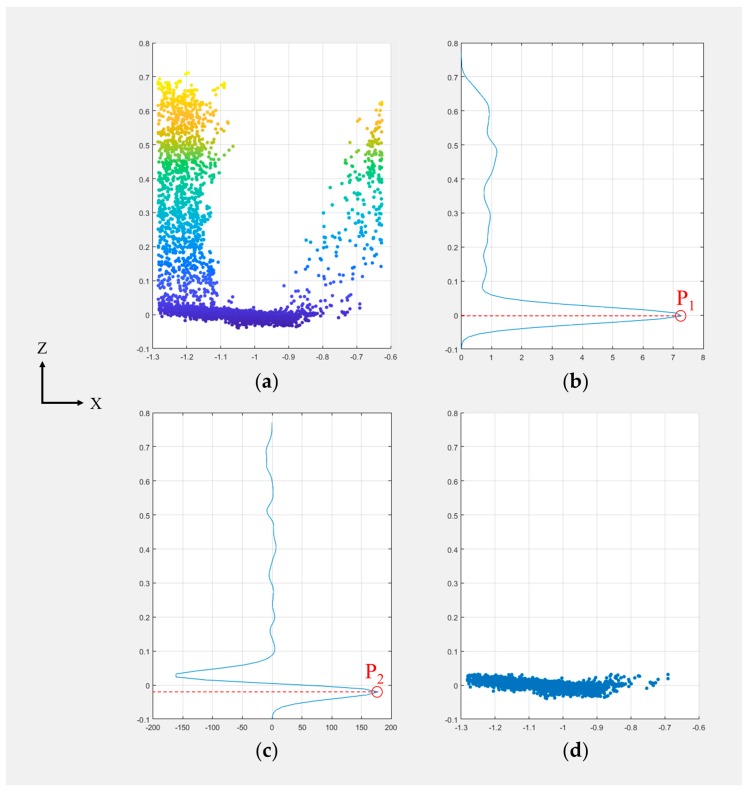
An example of extracting a refined alleyway point cloud: (**a**) point cloud of ground before cleaning; (**b**) point cloud kernel density in the Z dimension; (**c**) first derivative of the kernel density; (**d**) point cloud of ground after cleaning.

**Figure A4:**
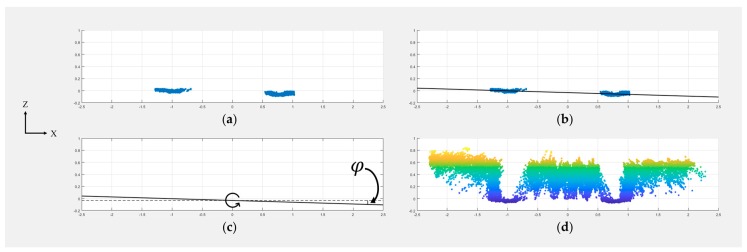
An example of X-Z plane rotation correction: (**a**) point cloud of ground before rotation; (**b**) linear curve fitted to ground points on the X-Z plane; (**c**) rotation of points on the X-Z plane by the angle φ; (**d**) point cloud after rotation.

**Figure A5:**
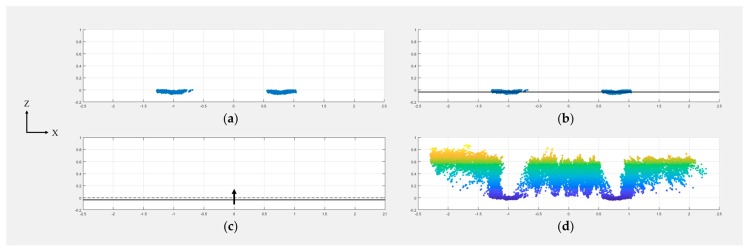
An example of ground baseline correction: (**a**) point cloud of ground before shifting; (**b**) the mean in the Z dimension; (**c**) points on the X-Z plane shifted by the offset; (**d**) point cloud after shifting.

**Figure A6:**
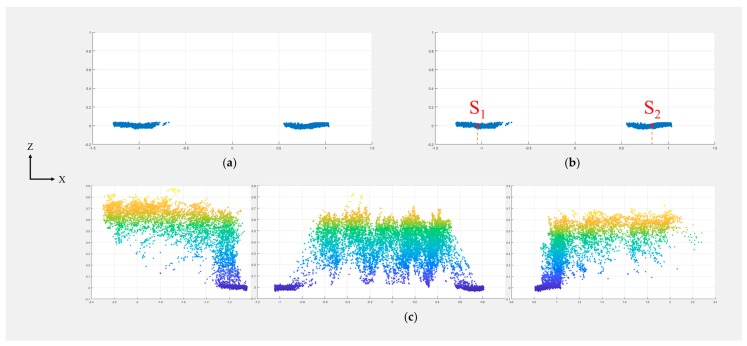
An example of splitting a point cloud: (**a**) point cloud of ground after rotation and shifting; (**b**) the mean in the X dimension for each side; (**c**) point cloud of each plot after splitting.

## Appendix A

Steps for LiDAR point cloud pre-processing are explained here in detail.

### Appendix A.1. Read Files

Three csv files containing XYZ coordinates of three-point clouds generated at the same measurement were read and combined back as one-point cloud (Figure 5).

### Appendix A.2. Y-Z Plane and X-Y Plane Rotation Correction

One reasonable assumption is that the points of a point cloud without the slanting issue should be evenly distributed along the Y dimension considering plants with the same genotype should have similar heights. A linear least-squares curve was fitted to the Y-Z plane (Figure A1b). The slope of the fitted curve was then converted to an angle θ in radiance through the relationship:

θ = arctan (slope). (A1)

The point cloud was finally rotated clockwise by the angle θ (Figure A1c). The rotation center could be set at any point, as later the point cloud will be repositioned in the Z dimension.

A similar procedure was also undertaken for the X-Y plane, which could be skipped as the slanting issue of point clouds on X-Y plane was minimum.

### Appendix A.3. X-Z Plane Rotation Correction

As the point distribution along the X-dimension could not be assumed to be even because points were representing plants with different genotypes, linear curve fitting couldn’t be directly applied to the X-Z plane. The method proposed here was to find the rotation angle by finding the average Z value difference between the ground points of two alleyways.

The points were first sorted by their X values so that the line graph of the points on the X-Z plane would have a horizontal curve (Figure A2c). Then, a moving average filter with a 0.05-m span was applied to smooth the curve (Figure A2d). Since the FOV of LiDAR could cover two alleyways, the trend of the curve typically had four abrupt changes in the Z dimension as lasers would scan from the canopy to the ground and back to the canopy twice. After finding the position of the four most significant changes (Figure A2e), points with X values smaller than C_1_, larger than C_4_, or between C_2_ and C_3_ were deleted so that the portion of point cloud that contained two alleyways in the X dimension was extracted (Figure A2f).

The point cloud containing two alleyways (Figure A2f) was separated into left and right alleyway point clouds using the border of X = 0. The non-ground points of two alleyway point clouds were further removed using the procedure explained below.

The kernel density in terms of Z values of the alleyway point cloud was first estimated (Figure A3b). As the points of ground were typically clustered at the bottom of the Z axis, a dominant peak P_1_ could be observed from the kernel density graph, which was also the first peak in the Z axis direction. The first derivative of the kernel density curve was calculated (Figure A3c). Assuming ground points follow a normal distribution in the Z dimension, the first peak P_2_ of the first derivate curve in the Z axis direction would be the inflection point of the normal distribution, and the distance between P_1_ and P_2_ would be one standard deviation of the distribution. For a normal distribution, the range μ ± 2σ includes about 95.45% of the values. Here a threshold of μ + 2σ on the Z axis was used to separate non-ground points from ground points, where μ is P_1_ and σ is P_1_ − P_2_, and points with Z values larger than the threshold were deleted (Figure A3d).

After combining refined left and right alleyway point clouds (Figure A4a), a linear least-squares curve was fitted to the combined alleyway point cloud on the X-Z plane (Figure A4b), and the point cloud with the Y-Z and X-Y plane rotation correction performed (Figure A2a) was rotated by the angle φ, which was derived from the slope of the fitted curve (Figure A4c).

### Appendix A.4. Ground Baseline Correction

The logic of the X-Z plane rotation correction was again executed on the point cloud with the X-Z plane rotation correction already performed (Figure A4d) to extract the rotated and refined alleyway point clouds (Figure A5a). The average Z value of the alleyway point cloud was calculated (Figure A5b), and the Z values of the whole point cloud (Figure A4d) were adjusted so that the average Z value of the alleyway point cloud would be located at 0.

### Appendix A.5. Split Point Cloud

The mean X values S_1_ and S_2_ of two alleyway point clouds were calculated (Figure A6b) and used as the border between different plots to split the point cloud (Figure A6c).
